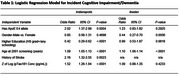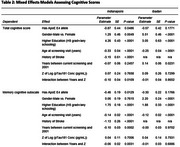# Longitudinal analysis of plasma *p*‐Tau181 in African and African American populations from the Indianapolis‐Ibadan Dementia Cohort

**DOI:** 10.1002/alz70856_107656

**Published:** 2026-01-09

**Authors:** Kristen A. Russ, Kathleen A. Lane, Sujuan Gao, Frederick W. Unverzagt, Hugh C Hendrie, Tatiana M. Foroud, Jeffrey L. Dage

**Affiliations:** ^1^ Indiana Alzheimer's Disease Research Center, Indianapolis, IN, USA; ^2^ Indiana University School of Medicine, Indianapolis, IN, USA

## Abstract

**Background:**

The Indianapolis‐Ibadan Dementia Project (IIDP) is a longitudinal epidemiological study that evaluated subjects in Indianapolis, Indiana and Ibadan, Nigeria for prevalence and incidence of cognitive decline and dementia between 1991‐2012. Plasma collected from the 2001 wave is currently stored at the National Centralized Repository for Alzheimer's Disease and Related Dementias (NCRAD). We analyzed the IIDP plasma to assess whether the ability of *p*‐Tau181 to predict disease differed between subjects in these two geographically, culturally, genetically, and environmentally disparate sites.

**Method:**

Plasma collected from study participants was analyzed for Alzheimer's disease biomarkers using the Quanterix Simoa HD‐X pTau181 v2 Advantage kits. The mean and standard deviation of the log transformed biomarker data using all subjects with normal cognition from 2001 were used to standardize the biomarker prior to statistical analysis. For this analysis, participants with normal cognition during the 2001 wave and at least one follow‐up evaluation were included (*N* = 755 African Americans; *N* = 864 Nigerians). Incident cognitive impairment status was determined using the last wave the subject was assessed.

**Result:**

Using logistic regression models, *p*‐Tau181 was shown to predict incident cognitive impairment in the African American population (Odds Ratio (OR)=1.52, *p* < 0.0001) but not in the Ibadan population (OR=1.09, *p* = 0.42) adjusting for age, sex, education, APOE4 genotype and history of stroke. The results from the logistic regression model for incident cognitive impairment/dementia can be seen in Table 1. Mixed effects models assessing cognitive scores showed significant interactions between time and *p*‐Tau181, indicating that higher *p*‐Tau181 is associated with greater decline for total cognitive score (*p* = 0.0109) and memory cognitive subscale (*p* = 0.0031) only in the Indianapolis African American population (Table 2).

**Conclusion:**

While geographic location has been seen to influence APOE genotype risk, similar investigations of AD biomarkers has been limited. Our data suggest there are differences in etiology that drives cognitive impairment between the African Americans in Indianapolis and the Africans in Ibadan. Additional analyses must be done to determine what drives these differences and the most effective biomarkers or biomarker combinations for differing populations.